# Osteomyelitis in Pig Carcasses at a Portuguese Slaughterhouse: Association with Tail-Biting and Teeth Resection

**DOI:** 10.3390/ani14121794

**Published:** 2024-06-15

**Authors:** Pedro Teiga-Teixeira, Melissa Alves Rodrigues, Dina Moura, Eduardo Teiga-Teixeira, Alexandra Esteves

**Affiliations:** 1 General Directorate of Food and Veterinary Affairs of the Northern Region, Barcelinhos, 4755-060 Barcelos, Portugal; dina.moura@dgav.pt; 2 School of Agricultural and Veterinary Sciences, University of Trás-os-Montes and Alto Douro, 5001-801 Vila Real, Portugal; anamelissa4@gmail.com; 3 Faculty of Sciences, University of Porto, 4169-007 Porto, Portugal; joao.edu.t@hotmail.com; 4 Department of Veterinary Sciences, University of Trás-os-Montes and Alto Douro, 5001-801 Vila Real, Portugal; alexe@utad.pt; 5 Animal and Veterinary Science Centre (CECAV), Associate Laboratory for Animal and Veterinary Sciences (AL4AnimalS), University of Trás-os-Montes and Alto Douro, 5001-801 Vila Real, Portugal

**Keywords:** osteomyelitis, tail-biting, teeth resection, meat inspection, pigs, animal welfare

## Abstract

**Simple Summary:**

Osteomyelitis is a significant cause of economic losses in swine production. The present study analysed the data collected in a Portuguese abattoir for finishing pigs. Total carcass condemnations, tail-biting lesions, husbandry invasive procedures, and several other factors were considered. The main cause of total carcass condemnation was osteomyelitis, with most cases being present in the skull and posterior region. Osteomyelitis in the skull was mainly located in the mandibular bone. Pigs with clipped teeth were associated with a higher occurrence of osteomyelitis. Tail-biting lesions and pleurisies were also linked to a higher occurrence of osteomyelitis. Improvements in veterinary inspection should be made to better correlate welfare and management factors with osteomyelitis in pig carcasses.

**Abstract:**

Osteomyelitis is the leading cause of total carcass condemnation in finishing pigs in Portugal, causing significant economic losses in swine production. The present study sought to determine a possible link between osteomyelitis in pig carcasses, pre-slaughter factors, and concomitant post-mortem inspection findings. For this purpose, meat inspection data were collected from 100,489 finishing pigs slaughtered in a northern Portuguese abattoir. Information regarding total carcass condemnation, slaughter season, origin, sex, tail-biting lesions, and husbandry invasive procedures (tail docking and teeth resection) was collected. The main cause of total carcass condemnation was osteomyelitis (61.03%). A total of 36.16% of osteomyelitis cases were present in the anterior region and 52.20% in the posterior region. In the anterior region, 94.78% of osteomyelitis cases were in the mandibular bone. Pigs with clipped teeth and carcasses with pleurisies were associated with a higher occurrence of osteomyelitis (*p* = 0.00262 and *p* < 0.0001, respectively). Second- and third-grade tail-biting lesions were also linked to a higher occurrence of osteomyelitis (*p* = 0.00128 and *p* < 0.0001, respectively). Slaughter inspection and monitoring procedures should be revised to better assess welfare factors and correlate management practices with the occurrence of osteomyelitis in pig carcasses.

## 1. Introduction

Osteomyelitis is an inflammatory disease affecting the bone and bone marrow across various animal species [1,2]. Osteomyelitis is a bone abscess, a deforming inflammatory process with an accumulation of pus, often associated with bacterial infections due to skin lesions during the swine production process [3,4,5]. This disease is often caused by infection by pyogenic organisms, namely, *Trueperella pyogenes*, *Streptococcus* spp., and *Staphylococcus* spp. [1,4,5]. In finishing pigs, osteomyelitis causes a high economic impact [1,3,5,6,7,8]. As cases of acute and purulent osteomyelitis are commonly linked with pyemia [3,5], the carcass of the animal must be deemed unfit for human consumption according to EU meat inspection regulations [3,5,9]. In Portugal, vertebral osteomyelitis is determined to be the main cause of economic impact in finishing pigs at slaughter [5,6]. Osteomyelitis is frequently challenging to diagnose in farm animals, and treatment is not usually feasible for economic reasons [2].

Veterinary inspection at the slaughterhouse focuses primarily on the removal from the food chain of meat and organs unfit for human consumption [10]. However, it also provides vital information regarding slaughtered animals’ health and welfare conditions [8,10,11]. The post-mortem inspection findings of slaughtered pigs usually reflect problems due to disease or poor welfare [11]. Such data can be useful for farms to detect health and welfare problems and improve animal production. [10,11]. Both consumers and stockholders have been demanding developments on more efficient animal welfare controls from farm to slaughter over the years [12,13]. Several studies have approached the impact of farm management factors such as weather conditions, husbandry practices, invasive procedures, and animal handling on the welfare of the animals and, consequentially, their meat quality and safety after slaughter [6,10,11,12,13,14,15,16,17,18]. However, few of these studies have focused on the impact of such factors on the occurrence of osteomyelitis in pig carcasses. Although a link to welfare mismanagement has been determined, research into the aetiology of osteomyelitis remains scarce [2].

Tail-biting is a behavioural disorder that continues to be a major problem in the swine production industry, with tail docking remaining an ineffective solution to the problem [16,19,20]. Much research has centred on the negative consequences of tail-biting on animal welfare and production costs [19,20,21,22,23,24,25,26]. The multi-factorial syndrome is linked to decreased weight gain, increased use of medication, increased transmission of disease, higher production costs and, ultimately, total carcass condemnations at slaughter [19,20,21]. Tail lesions promote the risk of disease, which can affect meat safety [16,19]. A tail with open wounds allows bacteria to enter the bloodstream, causing the dissemination of pathogens into several organs and tissues of the animal [20]. The spread of infection can result in the formation of abscesses, lung lesions, arthritis, and osteomyelitis in the caudal and thoracic vertebrae of the pig carcass [19,20,21]. At the slaughterhouse, tail-biting lesions are often associated with generalised carcass infections, leading to total carcass condemnations [16,20,26]. Lung lesions are quite prevalent in severe tail-bitten carcasses [20,21,26,27]. As tail-biting tends to be more common in farms where animals are more prone to stress and disease, concomitant respiratory findings, such as pleurisies and pneumonia, are also usually detected during post-mortem inspection at the abattoir [27].

Teeth resection is an invasive procedure still commonly used in piglets [28]. This procedure is highly discouraged in the European Union and is only permitted in severe welfare situations [29,30]. Nevertheless, it continues to be a routine practice in pig farms [29,31]. This procedure is performed on the first days of the piglets’ life to help prevent lesions caused by littermates and lesions to the sow’s teats and ultimately improve overall performance [28]. The eight “needle teeth” (the incisor and canine tooth on each side of the upper jaw and mandible) are either clipped with cutting pliers or ground with a rotating grinder [32,33]. The benefits of teeth resection are highly disputed, as skin lesions and growth rates between tooth-resected and intact teeth pigs vary considerably [28]. Teeth resection causes short- and long-term stress to the animal [29,34]. The damage of the highly innervated dental pulp during the procedure causes severe pain [29]. The procedure can also cause damage to the gums and roots of the teeth, such as dental fractures, gingivitis, pulpitis, dental pulp exposure, snout swellings, and abscess formation [29,32,34,35,36]. Bacteria gain access to the bloodstream via teeth lesions, and infection can be transmitted to other body parts [36]. Despite this, no association has ever been determined between teeth-resected pig carcasses and the presence of osteomyelitis.

This study aimed to obtain more information regarding the prevalence of osteomyelitis in finishing pig carcasses and determine the areas most affected by infection. Statistical analyses were performed to determine a possible association between osteomyelitis in pig carcasses and tail-biting lesions, teeth resection, pre-slaughter factors, and other concomitant post-mortem inspection findings.

## 2. Materials and Methods

**Figure 1:**
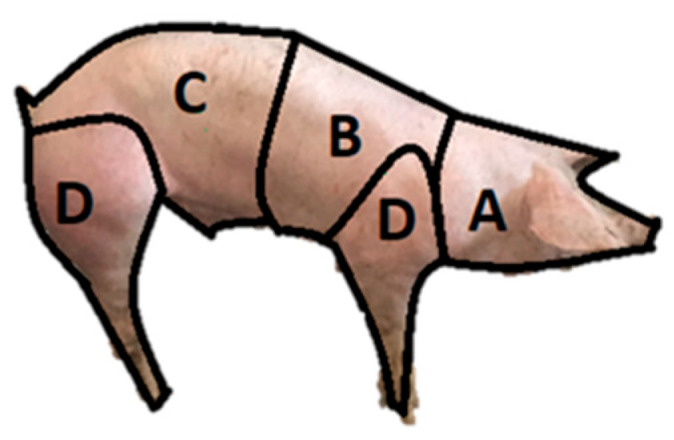
Pig model with divided areas: (**A**) anterior area, including the skull and cervical vertebrae; (**B**) thoracic area, including thoracic vertebrae, ribs, and sternum; (**C**) posterior area, including lumbar and coccygeal vertebrae, coxae, and sacrum; (**D**) limbs.

**Figure 2:**
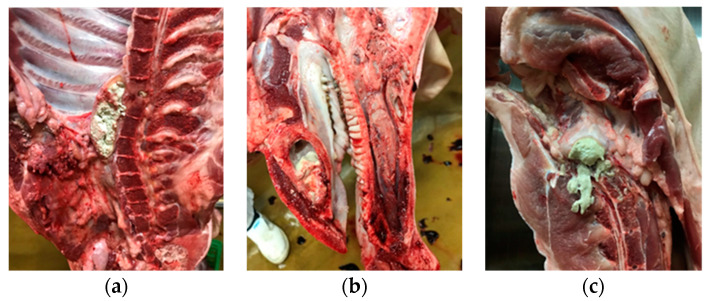
Osteomyelitis in different locations of the carcass: (**a**) thoracic area; (**b**) anterior area; (**c**) posterior area.

From 1 January 2020 to 1 May 2022, meat inspection data from 100,489 slaughtered finishing pigs were collected at one pig abattoir in the northern region of Portugal. A finishing pig is a pig that reaches its market slaughter weight [11], usually at 6 to 8 months of age. Total condemnations (TCs) of pig carcasses, their respective causes, and concomitant lesions were recorded during the referred period. In cases of carcasses condemned for osteomyelitis (CCOs), the affected region was recorded. A map model of the pig carcass dividing it into areas was created (Figure 1). Figure 2 shows the presence of osteomyelitis in different locations on the carcass.

The following pre-slaughter factors were recorded: slaughter season (spring, summer, autumn, and winter), animal origin (Portugal, Spain, France), and sex (female, male).

Information related to concomitant post-mortem inspection findings, such as cardiorespiratory inflammatory lesions (pneumonia, pleurisies, and pericarditis), signs of invasive husbandry procedures (tail docking and teeth resection) and tail-biting lesions, was collected. The presence and type of teeth resection were gathered during the post-mortem inspection or by direct communication with the respective pig farmers.

**Figure 3:**
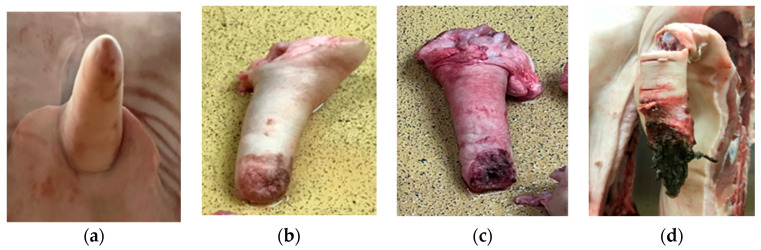
Grading of tail lesions: (**a**) no lesion; (**b**) light lesion; (**c**) severe lesion; (**d**) severe lesion with clear infection.

A protocol (Figure 3) for tail lesion grading based on vom Brocke et al. (2019) was applied [19].

Descriptive, univariate, and logistic regression analyses were performed using R version 4.1.0 software. Logistic regression was performed to model the probability of osteomyelitis occurrence based on pre-slaughter factors and concomitant post-mortem lesions.

The final logistic regression models presented in this work were derived through a rigorous process of variable selection using backward elimination using backward selection, i.e., the exclusion of less significant variables in the Wald test and having the most inconsistent confidence intervals. Model improvement steps included using likelihood ratio tests and the Akaike information criterion (AIC). Assessment of model assumptions, e.g., independence of observations, the existence of multicollinearity, and outlier analyses, were performed for all final models. Additionally, multiple interaction terms were explored between the independent variables. Variables with a *p*-value of ≤0.05 were considered significant. *p*-values below 0.001 were considered highly significant, and between 0.001 and 0.01 very significant. Odds ratios were calculated with 95% confidence intervals to quantify the magnitude and precision of estimated effects.

## 3. Results

### 3.1. General Descriptive Data Analysis

**Figure 4:**
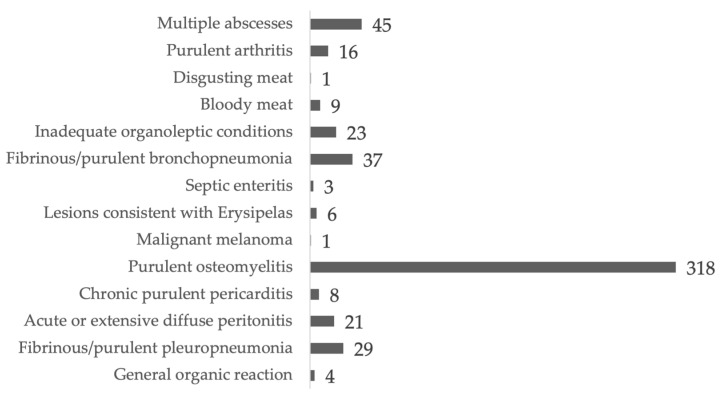
Absolute frequency of TCs according to the cause of the condemnation.

From 1 January 2020 to 1 May 2022, 100,489 finishing pigs were slaughtered, of which 521 (0.52% of the total slaughtered animals) were deemed unfit for human consumption. The main cause of TC was osteomyelitis (318 CCOs, representing 61.03% of TCs). Forty-five carcasses were condemned for multiple abscesses (8.64% of TCs), 37 for fibrinous/purulent bronchopneumonia (7.10% of TCs), 29 for fibrinous/purulent pleuropneumonia (5.56% of TCs), 23 for inadequate organoleptic conditions (4.41% of TCs), 21 for acute/extensive peritonitis (4.03% of TCs). Forty-eight other carcasses were condemned for other causes (9.21% of TCs) (Figure 4).

**Figure 5:**
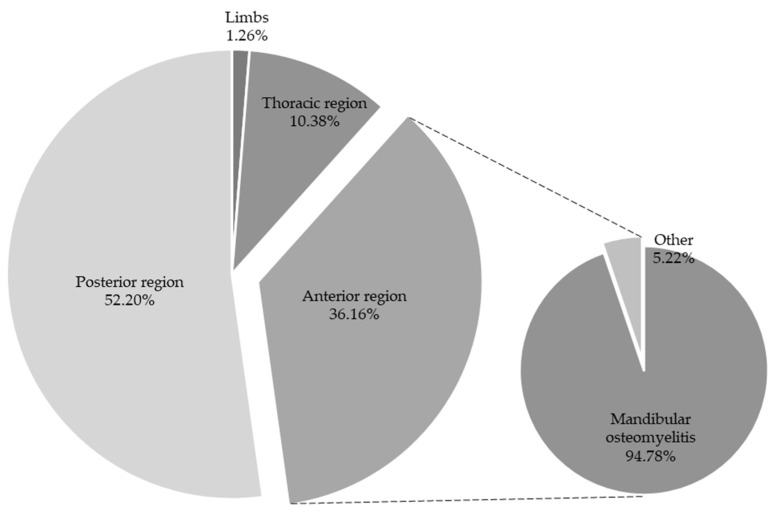
Location of osteomyelitis in CCOs (anterior region, limbs, thoracic region, and posterior region).

Regarding the 318 CCOs, 115 cases (36.16% of CCOs) were in the anterior region, 33 cases (10.37% of CCOs) were in the thoracic region, 166 cases (52.20% of CCOs) were in the posterior region, and 4 cases (1.25% of CCOs) were in the limbs (Figure 5). All cases of osteomyelitis in the posterior and thoracic region were in the vertebrae. Of the 115 CCOs in the anterior area, 109 cases were present in the mandibular bone, representing 34.27% of CCOs (Figure 5).

One hundred and eleven cases of CCOs were recorded in the spring (34.90% of CCOs), 69 cases in the summer (21.69%), 66 cases in the autumn (20.75%), and 72 cases in the winter (22.64%) (Table 1).

Two hundred and forty-five cases (77.04% of CCOs) of CCOs were originally from pigs reared in Portuguese farms, 59 cases (18.55%) were from Spanish farms, and 14 cases (4.40%) were from French farms (Table 1).

Regarding sex, 220 CCOs (69.18% of CCOs) were detected in females and 98 cases (30.81%) in males (Table 1).

Regarding cardiopulmonary post-mortem inspection findings in CCOs, 115 carcasses presented pleurisy (36.16% of CCOs), 32 presented pneumonia (10.06% of CCOs), and 52 presented pericarditis (16.35% of CCOs) (Table 2).

Twenty-six CCOs presented grade 1 tail-biting lesions (8.18% of CCOs), 26 presented grade 2 tail-biting lesions (8.18% of CCOs), and 56 presented grade 3 tail-biting lesions (17.61% of CCOs). Two hundred and ten (66.04%) CCOs presented no tail-biting lesion (Table 2).

Two hundred and seventy-nine cases (87.73%) of CCOs presented teeth resection (clipped or ground teeth), while 39 cases (12.26%) had intact teeth. Regarding the method of teeth resection, 226 (71.07%) animals were clipped, and 53 (16.67%) were ground (Table 2).

Further descriptive analysis showed the relationship between the osteomyelitis location in CCOs and the tail-biting lesion grade (Table 3) and the presence and type of teeth resection (Table 4).

CCOs in the anterior region presented six cases (1.88%) of grade 3, seven cases (2.20%) of grade 2, and six cases (1.88%) of grade 1 tail-biting lesions. CCOs in the thoracic area presented three cases (0.94%) of grade 3, one case (0.31%) of grade 2, and four cases (1.26%) of grade 1 tail-biting lesions. Forty-six cases (14.46%) of grade 3, 17 cases (5.34%) of grade 2 and 16 cases (5.03%) of grade 1 tail-biting lesions were detected in CCOs in the posterior region. The limb regions only registered one case of grade 3 tail-biting lesion and one case of grade 2 tail-biting-lesion (Table 3).

CCOs in the anterior region presented 92 cases (28.93% of CCOs) of clipped teeth, 15 cases (4.71%) of ground teeth, and 8 cases (2.51%) of intact teeth. CCOs in the thoracic region presented 18 cases (5.66%) of clipped teeth, 9 cases (2.83%) of ground teeth and 6 cases (1.88%) of intact teeth. CCOs in the posterior region presented 113 cases (35.53%) of clipped teeth, 28 cases (8.80%) of ground teeth, and 25 cases (7.86%) of intact teeth (Table 4).

### 3.2. Univariate Analysis of Carcasses Condemned for Osteomyelitis

Pearson’s Chi-squared tests were performed to determine the relationship between the occurrence of CCOs and the presence or absence of concomitant conditions, such as pleurisy, pneumonia, tail-biting lesions, and the presence and method of teeth resection.

A significant relationship (*p* ≤ 0.05) was found between the cause of condemnation (osteomyelitis or other causes) and multiple independent variables, e.g., pleurisy classification (present or absent) (*p* < 0.0001), pneumonia classification (present or absent) (*p* < 0.0001), teeth resection type (*p* = 0.0001078), and tail-biting lesion grade (*p* < 0.0001).

The relationship between the location of the osteomyelitis in the carcass and the tail-biting lesions classifications was significant (*p* < 0.0001).

The univariate analysis determined no significant association (*p* > 0.05) between the location of osteomyelitis and the type of teeth condition (*p* = 0.0584).

### 3.3. Logistic Regression Analysis

Animals slaughtered during autumn (*p* = 0.046, OR = 1.94) or summer (*p* = 0.007, OR = 0.48) presented a higher occurrence of osteomyelitis, translating into 94.37% and 48.77% (respectively) more odds of presenting osteomyelitis than those slaughtered in spring (*p* = 0.049, OR = 1.94) (Table 5).

Pleurisy was highly significant to the occurrence of osteomyelitis (*p* < 0.0001, OR = 0.30) (Table 5).

Carcasses with grades 2 and 3 tail-biting lesions were associated with a higher occurrence of osteomyelitis (*p* = 0.00128 and *p* < 0.0001, respectively), representing 471% and 1714%, respectively, more odds of presenting osteomyelitis (OR = 5.71 and OR = 18.14, respectively) than pigs without tail-biting lesions (Table 5).

Pigs with clipped teeth were associated with a higher occurrence of osteomyelitis (*p* = 0.00262), with 171% more odds of being associated with osteomyelitis (OR = 2.71) (Table 5).

## 4. Discussion

Osteomyelitis was by far the most registered cause for total condemnation of pig carcasses in our study (61.04% of TCs), followed by multiple abscesses (8.63% of TCs) and bronchopneumonia (7.10% of TCs). This is in accordance with similar studies on the subject [6,37]. The most common carcass location of osteomyelitis was in the posterior and thoracic vertebrae (199 cases), amounting to a considerable 62.57% of total CCOs. This aligns with Vieira-Pinto et al. (2020), as they considered vertebral osteomyelitis the main cause of the total condemnation of pig carcasses in Portugal [5]. One surprising result was the high number of osteomyelitis cases found in the anterior region of the pig carcass, particularly in the mandibular area. To our knowledge, no such finding has ever been reported in other similar studies. A possible connection to teeth management was analysed and discussed further. It is nevertheless evident that CCO represents a negative economic impact on the Portuguese swine industry, and further studies should be made to combat the lack of sufficient research into the topic.

Čobanović et al. (2020) stated that the most extreme temperature seasons (winter and summer) are particularly harmful to the welfare and health of pigs, primarily during transport, leading to decreased meat quality [14]. However, in this study, spring (mildly warm climate) exhibited the highest rates of CCOs (34.90%), followed by winter (22.64%), summer (21.69%), and autumn (20.75%). This result could be explained by the fact that the abattoir where the study was performed increased its slaughter rate during March, April, and May, escalating the odds of more reported TCs. Furthermore, the TC findings only pertain to condemnations recorded during the post-mortem inspection stage, neglecting to account for transport losses registered at the reception stage. Transport factors such as distance and transport densities were also not registered. These factors are usually substantially influenced by season [15,38]. Despite the higher percentage of CCOs in the spring, no statistical significance to the occurrence of CCOs was determined. However, a slight statistical significance was determined in autumn and summer.

Portuguese slaughtered pigs had the highest rate (77.04%) of CCOs in this study, reaching 245 TCs, followed by Spanish pigs with 59 TCs (18.55%) and French pigs with 14 TCs (4.40%). Different origins could mean different management practices and travel times, which could have different rates of impact on animal welfare and CCOs. However, this discrepancy is easily explained, as most batches that arrived at the slaughterhouse were of Portuguese origin. Two Spanish batches would arrive for slaughter twice a week, and batches from France would arrive only once monthly. Statistically, it is fair to assume that Portuguese farms would have had the highest rates of TCs and CCOs in this study. No relevant link was determined between the origin and CCO.

Many studies have emphasised the impact of sex on animal welfare, with male pigs being more prone to injuries, stress, tail-biting lesions, and ultimately, more post-mortem carcass findings [6,16,21,26,39]. However, our results showed that 220 CCOs were female, amounting to 69.18% of CCOs. The male CCOs only reached 98 cases (30.81% of CCOs). This outcome is unsurprising, as most pig batches processed at the abattoir were predominantly female. Although non-castrated males tend to have more post-mortem lesions due to higher aggressive behaviour [12], we decided not to divide the males regarding the presence of castration, as most pig batches consisted of castrated animals. Despite variations in physiology, behaviour, and post-mortem findings between sexes [6,12,26], our study did not uncover any direct correlation between sex and CCO and other inspection findings. In Portugal, finishing pigs are 6 to 8 months of age when they are subjected to slaughter. Such a short period of life does not allow animals much time to express sex-related behaviours after reaching sexual maturity. This fact, along with the low number of slaughtered males, can help explain the lack of correlation in our study between sex and post-mortem inspection findings. In addition to sex, one interesting factor for this study would be carcass weight. Chronic disease causes decreased weight gain in finishing pigs [7]. These growth-retarded animals stay longer in the production phase and are continually medicated, leading to higher production costs [7]. Compared with healthy pigs, growth-retarded pigs are more prone to lesions and total carcass condemnations due to abscesses, cachexia, arthritis, pleuritis, pneumonia, and osteomyelitis [7]. Unfortunately, the abattoir where the study occurred did not weigh condemned carcasses. Therefore, a link between CCO and decreased production growth rate could not be determined.

Respiratory problems such as pleurisies and pulmonary consolidations are persistent post-mortem findings in pig carcasses at slaughter [19,20,21,26,27,40]. In our study, 66 TCs (12.66% of TCs) were due to severe respiratory findings (bronchopneumonia and pleuropneumonia). Several studies have focused on finding a link between respiratory findings and other inspection findings, such as skin lesions, tail-biting lesions, arthritis, and pericarditis [17,27,41]. The present study revealed that concomitant cardiorespiratory post-mortem findings were prevalent in CCOs. Pleurisy was present in 36.16% of CCOs. Signs of pneumonia (10.06%) and pericarditis (16.35%) were also present. Furthermore, a solid statistical significance (*p* < 0.0001) was found between pleurisies and CCOs. Pleurisies are commonly related to health and welfare problems during the pre-slaughter phase [27] and can be related to health problems leading to the occurrence of osteomyelitis. Monitoring post-mortem respiratory findings can thus help obtain valuable information about the health and welfare of pigs [27] and form a link to several causes of TCs, such as osteomyelitis.

Due to tail docking being performed in 100% of the slaughtered animals used for this study, the focus was redirected to tail-biting lesions in the carcasses. Tail-biting may result in significant economic losses in the swine industry due to secondary infections, such as osteomyelitis [26]. Severe tail-biting lesions are linked to increased TCs of pig carcasses, lung lesions, and decreased carcass weight [13]. The present study found a significant association between severe levels of tail-biting (grades 2 and 3) and CCO. It was determined that carcasses with grades 2 and 3 tail-biting lesions had great influence (471% and 1714% more chances, respectively) on the occurrence of osteomyelitis. Tail lesions linked to advanced degrees of infection and necrosis are believed to be related to osteomyelitis [26]. This is supported by the high percentage of CCOs with grade 2 or 3 tail-biting in the study. A lesion in the tail may promote the dissemination of pyogenic bacteria from the skin and environment via blood and lymph, resulting in abscesses in the adjacent tissue and osteomyelitis in the vertebrae [26,27]. It is logical to assume that the infection in tail-biting lesions may spread to adjacent locations, such as the sacral and coccygeal vertebrae, leading to osteomyelitis [5]. Despite the clear relationship between the location of the osteomyelitis and the grade of tail-biting lesions (*p* = 0.00128 in grade 2 tail-biting lesions and *p* < 0.0001 in grade 3 tail-biting lesions), it was not possible to find a statistical significance between tail-biting lesions and osteomyelitis in the posterior location of the carcass. Still, it is evident that most of the TCs due to osteomyelitis were related to osteomyelitis in the posterior region. While only 9.08% of CCOs in the anterior region, thoracic region, and limbs presented concomitant tail-biting lesions, the same post-mortem inspection finding was found to be more prominent in CCOs located in the posterior region (24.84%). This supports the conclusion that tail-biting in pigs is linked with significant carcass losses due to osteomyelitis [16,20,26]. TCs with no tail-biting lesions also presented a substantial number of CCOs in the anterior region (96 cases), thoracic region (25 cases), and posterior region (87 cases). Although the dissemination of infection will likely happen in pigs with tail-biting lesions, other points of entry can also provide access for pathogens to the animal’s bloodstream [20]. Infections can develop and disseminate through bites in other body regions [20]. Thus, despite tail-biting lesions being strongly associated with generalised infections such as osteomyelitis, other routes can also cause severe infection and, consequently, osteomyelitis in pig carcasses.

Another interesting result was the significant relationship between carcasses with resected teeth and CCO. Clipping teeth in piglets is a common practice in swine farms to reduce the incidence of facial skin lesions in piglets and lesions in the sows’ udders, although its effectiveness is questioned [28,32,42,43]. The resection is usually performed between the first and third days of the piglets’ life, as the animals already have four canines and four incisive teeth by birth [32,33,43]. Grinding the teeth is another option, although it can still cause short- and long-term pain to the piglet and tooth abnormalities [28,29]. Thus, despite some advantages, clipping may result in gingivitis, pulpitis, bacteremia, and arthritis [28,33,42]. Fu et al. (2019) described the impact of teeth clipping on the body surface temperature and concluded that there is a greater severity of pain when related to teeth clipping than tail docking [35]. Fractures, haemorrhages, and deep dental lesions can occur due to teeth resection [29,32,34]. Some studies recorded periapical abscesses with a major incidence on teeth subjected to clipping or grinding techniques [43,44]. Those are mainly associated with *Streptococcus* sp. infections [44]. Teeth resection has been continually discouraged in the European Union. The Commission Directive 2001/93/EC of 9 November 2001 states that “neither tail docking nor reduction of corner teeth must be carried out routinely but only where there is evidence that injuries to sows’ teats or other pigs’ ears or tails have occurred” [30]. Nonetheless, this practice is still frequently performed in many European countries [31]. In the present study, clipped teeth carcasses had a strong link (*p* = 0.00262) with the occurrence of osteomyelitis, something that, to our knowledge, has not yet been reported in any other research in a sanitary meat inspection context. The presence of clipped teeth in a carcass represents 171% more chances of it presenting osteomyelitis. Similar to the tail-biting cases [20,45], this may be explained by the risk of infection due to the painful lesions, which, in this case, were caused by the resection procedure [28]. As described previously, most osteomyelitis cases in the anterior region were present in the mandibular bone and, more specifically, in the mandibular symphysis. Due to its proximity to the dental roots, it is logical to consider the mandibular symphysis a probable location for a hypothetical infection dissemination caused by a deep tooth lesion [36]. The observation of mandibular osteomyelitis was only possible because the carcass preparation procedure at the specific abattoir determined that the head of the carcass must be cut sagittal along with the rest of the carcass. This would allow visual access to the interior of the mandibular symphysis. During the slaughter process, the pig carcasses are split longitudinally into two halves to facilitate veterinary inspection and cooling procedures [46]. However, no specific instruction is given to perform a sagittal splitting of the head of the carcass. From the author’s experience, splitting the head in half along with the carcass during slaughter depends mainly on the food business operator’s preference. Thus, potential cases of mandibular osteomyelitis can remain hidden from most veterinary post-mortem inspections. This might explain the fact that similar findings have not yet been described. Revision of carcass processing procedures during slaughter, at the European and national levels, can be beneficial in further detecting this finding.

## 5. Conclusions

The present study analysed data from finishing pig carcasses from three European countries (Portugal, Spain, and France) slaughtered in one Portuguese abattoir. We concluded that osteomyelitis is the main cause of total carcass condemnations in our study. Most osteomyelitis cases were found in the posterior vertebrae (lumbar and coccygeal) or in the mandibular symphysis. Osteomyelitis cases were strongly associated with pleurisies, tail-biting lesions, and clipped teeth. This suggests a solid link between osteomyelitis and farm management factors. The high prevalence of concomitant pleurisy in many osteomyelitis cases indicates a serious welfare problem. Monitoring concomitant respiratory lesions is essential in evaluating the animal’s health and welfare at slaughter. Our study reports a significant correlation between severe tail-biting lesions and osteomyelitis in pig carcasses. We reinforce the need for better monitoring and prevention of tail-biting from farm to slaughter. Our study also adds the impact of teeth clipping on meat safety, linking it to osteomyelitis, particularly in the mandibular bone. A thorough inspection of the skull should be added to post-mortem inspection protocols to identify hidden cases of mandibular osteomyelitis. Slaughter season, animal origin, and sex had no evident influence on the occurrence of osteomyelitis in this study.

Animal welfare mismanagement can have devastating effects on the quality and safety of meat. This study highlights the need for an all-encompassing welfare monitoring system, from production to carcass processing. A comprehensive ante-mortem and post-mortem inspection and a closer examination of welfare indicators at slaughter could be valuable in identifying recurring issues in swine production and preventing further financial losses due to osteomyelitis.

## Figures and Tables

**Table 1:** Frequencies of CCOs (318 cases) according to slaughter season, animal origin, and sex.

Variable	Classification	Abs. Freq. * (CCO)	Rel. Freq. ** (CCO)
Season	Spring	111	34.90%
	Winter	72	22.64%
	Autumn	66	20.75%
	Summer	69	21.69%
Origin	Portugal	245	77.04%
	France	14	4.40%
	Spain	59	18.55%
Sex	Female	220	69.18%
	Male	98	30.81%

* Absolute frequency; ** relative frequency.

**Table 2:** Absolute and relative frequency of CCOs (318 cases) according to the concomitance of cardiorespiratory lesions, tail-biting lesions, and the presence and method of teeth resection.

	Classification	Abs. Freq. *	Rel. Freq. **
Cardiorespiratory lesions	Pleurisy	115	36.16%
	No pleurisy	203	63.83%
	Pneumonia	32	10.06%
	No pneumonia	286	89.93%
	Pericarditis	52	16.35%
	No pericarditis	266	83.64%
Tail-biting	Tail-biting lesions grade 1	26	8.17%
	Tail-biting lesions grade 2	26	8.17%
	Tail-biting lesions grade 3	56	17.61%
	No tail-biting lesions	210	66.03%
Teeth resection	Intact	39	12.26%
	Clipped	226	71.06%
	Ground	53	16.66%

* Absolute frequency; ** relative frequency.

**Table 3:** Relationship between the location of CCO (318) and the tail-biting lesion grade (Pearson’s Chi-squared test: X-squared = 38.278, *p* = 1.554 × 10^−5^).

Location	Grade 1		Grade 2		Grade 3		Total Lesions		No Lesions	
	Abs. Rel. *	Rel. Freq. **	Abs. Rel. *	Rel. Freq. **	Abs. Rel. *	Rel. Freq. **	Abs. Rel. *	Rel. Freq. **	Abs. Rel. *	Rel. Freq. **
**Anterior** **region**	6	1.88%	7	2.20%	6	1.88%	19	5.97%	96	30.18%
**Limbs**	0	0.00%	1	0.31%	1	0.31%	2	0.62%	2	0.62%
**Posterior** **region**	16	5.03%	17	5.34%	46	14.46%	79	24.84%	87	27.35%
**Thoracic** **region**	4	1.25%	1	0.31%	3	0.94%	8	2.51%	25	7.86%

* Absolute frequency; ** relative frequency.

**Table 4:** Relationship between the CCO (classified by location of the osteomyelitis in the carcass) and the presence and type of teeth resection of 318 carcasses condemned for osteomyelitis.

Location	Intact Teeth		Clipped Teeth		Ground Teeth	
	Abs. Freq. *	Rel. Freq. **	Abs. Freq. *	Rel. Freq. **	Abs. Freq. *	Rel. Freq. **
**Anterior** **region**	8	2.51%	92	28.93%	15	4.71%
**Limb**	0	0.00%	3	0.94%	1	0.31%
**Posterior region**	25	7.86%	113	35.53%	28	8.80%
**Thoracic region**	6	1.88%	18	5.66%	9	2.83%

* Absolute frequency; ** relative frequency.

**Table 5:** Logistic regression results (residual deviance: 566.72 on 508 degrees of freedom).

	Estimate	Std. Error	*p*-Value	OR
(Intercept)	0.27	0.36	0.46	1.303544
Season winter (vs. spring)	0.15	0.29	0.60	1.164103
Season autumn (vs. spring)	0.66	0.33	0.04584 *	1.943729
Season summer (vs. spring)	−0.72	0.27	0.00716 **	0.487798
Origin France (vs. Portugal)	0.30	0.51	0.56	1.347112
Origin Spain (vs. Portugal)	0.35	0.37	0.35	1.416778
Pleurisy	−1.18	0.25	*p* < 0.0001 ***	0.306115
Pericarditis	−0.30	0.29	0.29	0.738054
Tail-biting lesion grade 1	−0.14	0.33	0.66	0.865161
Tail-biting lesion grade 2	1.74	0.54	0.00128 **	5.713419
Tail-biting lesion grade 3	2.90	0.62	*p* < 0.0001 ***	18.14192
Clipped teeth	1.00	0.33	0.00262 **	2.709421
Ground teeth	−0.50	0.44	0.26	0.609405

*** Highly significant (*p* < 0.001); ** very significant (*p* < 0.01); * significant (*p* < 0.05); OR, odds ratio.

## Data Availability

The data presented in this study are available on request from the corresponding author. The data are not publicly available due to privacy reasons.
